# Effect of Community Health Education Intervention on Prevalence and Pig Farmers' Knowledge, Attitudes, and Practices Related to Porcine Cysticercosis in Selected Areas of Tanzania

**DOI:** 10.1155/vmi/9929531

**Published:** 2025-11-24

**Authors:** Christina Wilson, Hezron Emmanuel Nonga, Robinson Hammerthon Mdegela, George Makingi, Dominik Stelzle, Ernatus Martin Mkupasi, Andrea Sylvia Winkler, Helena Aminiel Ngowi

**Affiliations:** ^1^Department of Microbiology, Parasitology and Biotechnology, College of Veterinary Medicine and Biomedical Sciences, Sokoine University of Agriculture, P.O. Box 3019, Chuo Kikuu, Morogoro, Tanzania; ^2^Department of Veterinary Medicine and Public Health, College of Veterinary Medicine and Biomedical Sciences, Sokoine University of Agriculture, P.O. Box 3021, Chuo Kikuu, Morogoro, Tanzania; ^3^Institute of Infectious Diseases and Tropical Medicine, LMU University Hospital, Munich, Germany; ^4^Center for Global Health, School of Medicine and Health, Technical University of Munich (TUM), Munich, Germany; ^5^Department of Neurology, TUM University Hospital, Technical University of Munich (TUM), Munich, Germany; ^6^Department of Community Medicine and Global Health, Institute of Health and Society, University of Oslo, Oslo 0318, Norway

**Keywords:** health education intervention, knowledge, mixed logistic regression, porcine cysticercosis, practices, the difference-in-differences (DID) method

## Abstract

Porcine cysticercosis (PCC) is a food-borne zoonotic disease prevalent in resource-poor rural communities with free-range pig management systems and low sanitation practices. Various prevention and control strategies have been implemented in Tanzania, with this study highlighting the importance of community-based health education in controlling the *Taenia solium* parasite. The study evaluated the effectiveness of community-based health education using a cocreated health education package (HEP) on the knowledge, attitudes, and practices (KAP) of smallholder farmers, as well as on the prevalence of PCC in Kongwa and Songwe Districts, Tanzania. A cluster-randomized health education intervention was conducted between June 2019 and December 2021, with pre- and postintervention evaluations of PCC prevalence and smallholder farmers' KAP. A baseline cross-sectional study was followed by health education training utilizing the HEP, which included brochures, booklets, and posters. The training was provided to trainers (TOT) who, in turn, trained the community. A commercial Ag-ELISA kit (apDia, Belgium) was used to identify circulating antigens in pig serum and determine the PCC prevalence both before and after the intervention. A total of 692 and 486 respondents participated in face-to-face interviews during the baseline and postintervention periods, respectively. Concurrently, 692 and 317 pigs were sampled during these periods. At baseline, the seroprevalence of PCC was 10.2% in the intervention group and 9.1% in the control group. Twelve months following the health education intervention, the study observed significant improvements in knowledge (*β* = 1.779, *p*=0.004), attitudes (*β* = 1.024, *p*=0.038), and practices (*β* = 0.719, *p*=0.023) among participants. Additionally, a reduction in PCC prevalence was observed—3.9% in the intervention group and 0.9% in the control group—though this difference was not statistically significant (OR = 0.70; 95% CI: 0.27–1.83; *p*=0.47). The reduction in PCC is a gradual process that likely requires a longer observation period to yield a measurable impact. The limited duration of follow-up may have constrained the study's ability to conclusively attribute the observed decline in PCC seroprevalence to the intervention. Evidence suggests that a comprehensive strategy targeting both intermediate and definitive hosts is essential. Therefore, future health education interventions should integrate mass drug administration for pigs and treatment of human taeniosis—an approach that could be effective in disrupting the lifecycle of *Taenia solium*.

## 1. Introduction

*Taenia solium* taeniasis*–*cysticercosis (TSTC) is a parasitic infection that affects both humans and pigs and is common in many developing countries [1, 2]. This foodborne parasite causes a significant global burden of disease, measured in disability-adjusted life years (DALYs) [3]. Pigs, as intermediate hosts, become infected by eating human feces containing the parasite's eggs or proglottids. Once inside the pig, the larvae develop into porcine cysticercosis (PCC) [4]. PCC is a major zoonotic disease that severely impacts pig production globally, with smallholder farmers being particularly affected. It lowers the market value of pigs and pork and has become a key obstacle to the trade of pigs and pork [5].

Humans act as the definitive hosts for *Taenia solium*, harboring the adult tapeworm that causes taeniasis [2]. Cysticercosis in humans occurs when viable eggs of *T. solium* are accidently ingested through contaminated food or water. Once inside the intestine, the eggs develop into oncospheres, which penetrate the intestinal wall and migrate to various tissues—including muscles and the central nervous system—where they form cysts. When cysts develop in the brain, the condition is known as neurocysticercosis [6, 7]. Neurocysticercosis is a major global cause of epilepsy, with a particularly high prevalence in endemic areas such as southern Tanzania [8–10]. The disease often goes undiagnosed, and even when detected, treatment—although available—can be complex and challenging to administer effectively [11–13].

The International Task Force for Disease Eradication (ITFDE) has designated PCC as an eradicable disease. However, the disease remains prevalent in Tanzania's northern, central, and southern highlands [14–16]. Multiple factors contribute to the persistence of PCC in endemic regions, including inadequate environmental sanitation, poor hygiene practices, poverty, suboptimal pig management, and limited public awareness regarding disease transmission [17]. Among these, insufficient education within communities stands out as a critical driver of behaviors that perpetuate transmission and spread of the disease [18–20]. Previous research conducted in Tanzania and other regions has explored various strategies for preventing and controlling TSTC. These include treatment of taeniasis in humans, treatment in pigs [21–24], and health education (HE) initiatives [25, 26]. Among these, HE stands out as a vital tool for raising awareness and promoting lasting improvements in pig management and hygiene practices—both of which are key to reducing the prevalence of PCC.

HE is recommended as a priority intervention, even in areas where other control measures are being implemented. Its success, however, depends on thoughtful design that takes into account the local context and adopts a One Health approach—integrating human, animal, and environmental health—while actively involving local communities throughout the process.

Despite its importance, only a few studies in Tanzania have evaluated the effectiveness of HE in the context of TSTC. These studies have shown promising results, including significant reductions in *T. solium* transmission, improved community knowledge and attitudes [25, 26], and better pig management practices [27]. However, earlier HE efforts were often limited by low levels of community engagement, with target groups not sufficiently involved in planning, development, or implementation. Additionally, many of these initiatives lacked long-term sustainability [25, 26].

This study implemented a health education package (HEP), consisting of brochures, booklets, and posters, codeveloped through active community participation to foster effective knowledge transfer and sustainable impact. The primary objective was to evaluate the effectiveness of this HE intervention in reducing the prevalence of PCC and improving the knowledge, attitudes, and practices (KAP) of pig farmers in the Kongwa and Songwe Districts of Tanzania.

## 2. Materials and Methods

### 2.1. Study Area

The study was carried out in PCC-endemic regions within the Kongwa and Songwe districts, located in Tanzania's central and southern highland zones, respectively. Detailed descriptions of these study sites have been previously provided by Wilson et al. [15].

### 2.2. Study Design, Sample Size, and Sampling

A cluster-randomized controlled trial was implemented to compare two groups: one receiving a HE intervention and a control group. Based on previous studies by Komba et al. [28] and Ngowi et al. [29] conducted in Mbozi and Kongwa districts, respectively, the baseline prevalence of PCC was estimated at 30% in both Kongwa and Songwe districts. Anticipating that the intervention would reduce seroprevalence to 15%, we aimed for 80% statistical power to detect a 50% reduction at a 95% confidence level.

The sample size was calculated by the formula *n* = [*Zα*√(2*pq*) − *Zβ*√*P*1*q*1 + *P*2*q*2]2/(*p*1–*p*2) 2 [30]. where *n* = estimated sample size, *Zα* = 1.96, confidence interval, *Zβ* = −0.84, *P*1, *P*2 estimated prevalence in the cross-sectional and posthealth education survey, respectively, *q*1 = 1 − *P*1, *q*2 = 1 − *P*2 (unaffected proportion of the population). Consequently, a sample size of *n* = 672 was needed for the investigation. During a randomized controlled field-intervention trial, all 130 villages (87 in Kongwa and 43 in Songwe District) were systematically screened for eligibility. This rigorous selection ensured the inclusion of independent and representative communities, thereby strengthening the validity of the study outcomes. Villages were selected using a probability proportional to size (PPS) sampling method, based on pig population data [31] and were required to meet the following inclusion criteria: a minimum of 20 pig-keeping households and sufficient accessibility for field teams [32]. Ultimately, 42 villages qualified; 28 from Kongwa and 14 from Songwe. Within the village, pig-keeping households were randomly selected from official village registers using randomly generated numbers in Microsoft Excel. One pig per household was examined to avoid intrahousehold clustering. Households were included based on the owner's willingness to participate and the presence of at least one pig aged 3 months or older. To meet the required sample size, 16 pigs and 16 households were targeted per village.

### 2.3. Data Collection

#### 2.3.1. Questionnaire and Observation Survey

Data were collected using a structured questionnaire developed through the Kobo Collect tool, which was installed on tablet devices for efficient digital entry. Before the main survey, the questionnaire was pretested in a village outside the designated study areas to ensure clarity and contextual relevance. The survey was administered through face-to-face interviews conducted with either the household head or an adult permanent resident of each selected household, both before and after the HE intervention. Interviews were carried out at the village office by a trained research assistant and the principal investigator.

The questionnaire comprised both closed-ended and open-ended items, addressing a range of topics including *Taenia solium* transmission and prevention, housing conditions, feeding practices, and general pig husbandry. Furthermore, a structured observation checklist was used to evaluate and verify hygiene and sanitation practices. Key indicators included the presence and condition of latrines, availability of handwashing facilities, and signs of open defecation.

Further observations focused on pig management and welfare, evaluating factors such as the number of pigs per household, type of management system, structural integrity of pig pens (including intact walls), and the presence of water troughs.

#### 2.3.2. Assessment of the Seroprevalence of PCC

Before sampling, data on each study pig—including age, sex, breed, and origin—were recorded based on farmer-reported history and direct observation during field visits. Approximately 5 mL of blood was drawn from the jugular vein using a sterile vacutainer needle or syringe and transferred into plain vacutainer tubes. The samples were then centrifuged at 1500 × *g* for 10 min to separate the serum. The recovered sera were aliquoted into 1.8 mL cryogenic vials and stored at −20°C until analysis. Circulating *Taenia solium* antigens were detected using a commercial cysticercosis antigen enzyme-linked immunosorbent assay (Ag-ELISA) kit (apDia, REF 650501, Belgium) [33], conducted at the CYSTINET-Africa Project laboratory, Sokoine University of Agriculture (SUA).

### 2.4. Randomization of Study Villages for Intervention and Control Groups

In allocating the villages to intervention and control groups, a stratified cluster randomization design was employed. We randomized the intervention to the village level to prevent information contamination. Within each district, villages were grouped into blocks (strata) with relatively similar baseline prevalence levels of PCC. The prevalence blocks used were (i) 0%, (ii) 4.5%–9.5%, (iii) 10.5%–33.3%. There were 10 villages in the first stratum with zero prevalence, 13 villages in the second stratum with a prevalence of 4.5% to 9.5%, and 19 villages in the third stratum with a prevalence of 10.5% to 33.3%: In each block (stratum), a simple randomization procedure was performed in Microsoft Excel to assign half of the village to each strata to either the intervention or control group. As a result, 21 villages were allocated to the intervention group (14 in Kongwa, 7 in Songwe) and 21 to the control group (14 in Kongwa, 7 in Songwe).

The overall baseline prevalence of PCC was approximately 10.4% (35 out of 336 pigs) in the intervention group and 9.0% (32 out of 356 pigs) in the control group, with no statistically significant difference between the two groups (*p*=0.5255). Thus, the two groups were well balanced in terms of their baseline levels of PCC. Randomization was based on PCC due to the fact that the study villages were selected based on pig population. Nevertheless, following randomization, the groups coincidentally remained well balanced in their baseline levels of both diseases. The group designated as the intervention receives HE, while the control group does not receive any HE.

### 2.5. Health Education Intervention

#### 2.5.1. Health Education Materials

Community health education was delivered using a specially designed package that included brochures, booklets, and posters. This HEP was developed based on key messages gathered from the target communities through a previous sociological study. This study was conducted by engaging the relevant communities and considering their level of awareness and risky behaviors related to *T. solium* infection, carried out in four districts: Mbulu, Mpwapwa, Mbinga, and Rungwe in Tanzania [34]. The posters, booklets, and leaflets were designed to be more visual than text-based, making the messages easier for illiterate pig farmers to understand. The poster, which illustrates the life cycle of the *T. solium* parasite, was used by the training the trainers (TOT) to show how both pigs and humans can develop cysticercosis and how humans can acquire taeniasis. The leaflets, containing information about the parasite's life cycle and prevention measures for taeniasis and cysticercosis, were distributed to participants after the training sessions. The booklets, which provide detailed information about the parasite's life cycle, WASH practices, and pig husbandry, were given to the TOTs. Seminars were held at churches, school classrooms, and village offices. The training covered several key topics, including the lifecycle of *Taenia solium*, strategies for preventing and controlling TSTC, improved hygiene practices, and water, sanitation, and hygiene (WASH) techniques. The training also emphasized best practices in pig management, such as building well-structured pig pens using locally available materials and providing feed from local food resources.

#### 2.5.2. Mode of Training Delivery

HE training was conducted from October to December 2020, in all treatment villages in Kongwa and Songwe Districts. The objective of the training was to improve smallholder farmers' knowledge and attitudes and reduce the risk behaviors associated with *T. solium* taeniasis/cysticercosis transmission. Before the start of the HE program, a meeting was conducted with the district and village officials to clarify the goals of the training. The baseline data on knowledge of the *T. solium* lifecycle, prevention, and WASH program for TOT were collected using the questionnaire. The training was given for 3 days, with the first 2 days dedicated to TOT, which included village and ward leaders, as well as livestock and human health specialists. The training for TOT was evaluated using the same questionnaire administered at pre-HE training. Then, three among the trained TOT with high scores were selected to participate in a single-day training of the chosen pig farmers. The approach was strategically designed to rapidly, cheaply, and exponentially upskill the workforce by developing local educators, ensuring the sustainability of HE in the study area beyond the study period [35]. The training was administered for about 5 h, and sections of the training included lectures, discussions (participant group discussions), questions and answer sections, and dissemination of brochures to the participants.

### 2.6. Post-HE Assessment of KAP

A follow-up survey was conducted 1 year after the community HE intervention, using the same data collection instruments as the baseline survey. To enhance the assessment, the principal investigator added seven additional questions to the baseline questionnaire. These questions aimed to determine the number of households that had ceased pig farming, the reasons for discontinuing pig farming, and whether any other programs or research initiatives focused on hygiene and pig management had been implemented in the study area during the study period.

### 2.7. Ethical Consideration

Ethical clearance was granted by the Tanzania National Institute for Medical Research (NIMR) Ethics Review Board (Ref. NIMR/HQ/R.8A/Vol.IX/2802) and the Vice-Chancellor of SUA (Ref. SUA/ADM/R.1/8/352). In Germany, the Klinikum rechts der Isar Ethics Committee at the Technical University of Munich approved the protocol (Permission No. 537/18 S-KK).

All participants provided written informed consent before enrollment. Village leaders offered verbal consent after a full briefing on the study objectives and farmers' right to opt out. Participant data were handled with strict confidentiality. After the trial concluded, the community HE program was extended to the original control group.

### 2.8. Data analysis

For cleaning and archiving, the survey data were downloaded from KOBO in excel format. The analysis was carried out using STATA (version 17; Stata Corp). Only HH with “full participants”—those who took part in the cross-sectional survey and the post-HE intervention survey—was considered in this analysis. The analysis was performed in three steps: (1) performance score of all questions in KAP was calculated, and the distinctions in the proportions in the correct responses on KAP towards PCC transmission and between farmers in the control and treatment groups were described using percentages. (2) Pearson's chi-square tests were used to determine whether KAP in the villages differed significantly between the baseline and postintervention periods for the treatment (intervention) and control groups. (3) The difference-in-differences (DID) method compares the changes in outcomes over time [31, 36]. Linear mixed model (LMM) DID analysis was also used to evaluate the effect of a community-based HE intervention on KAP. Other demographic characteristics of the study participants were controlled in the analysis. Additionally, mixed-effects logistic regression models were performed, assessing the changes in prevalence of PCC between intervention and control villages, considering the study design (clustering on village level and strata by district) by including random effects. Furthermore, included were fixed effects for pig sex and the age group of the pigs.

## 3. Results

### 3.1. Study Participants

**Figure 1:**
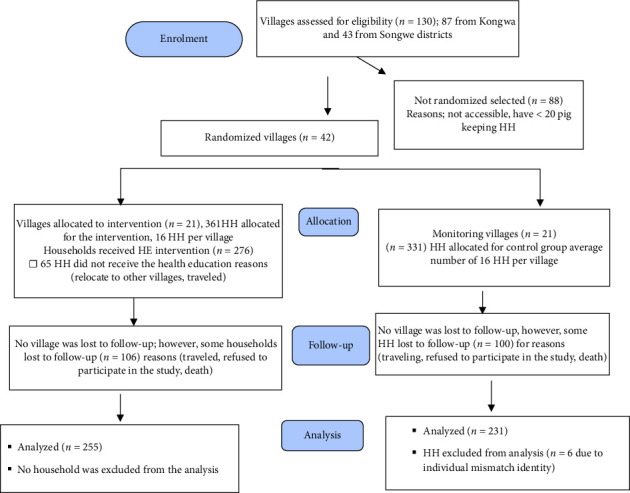
Flow of participants engaged in a community-based randomized health-education intervention trial conducted in Kongwa and Songwe districts, from 2019 to 2021.

The flow of participating villages and households during a community-based randomized HE intervention trial is shown in Figure 1. Following the training session, 276 participants (76%) in the treatment group, along with their community leaders, received informational leaflets. For pig farmers who did not attend the training, the leaflets were distributed by neighbors or local leaders. Of the farmers initially surveyed post-intervention, 492 (71%) completed the follow-up study—255 from the intervention group and 237 from the control group. However, the final analysis included only 486 participants due to a mismatch in individual identification (ID) numbers for six participants in the control group. During the follow-up survey, only 321 (66%) of the 486 households surveyed continued to keep pigs. Among the remaining households, 85 (52%) reported the death of their pigs due to African swine fever (ASF), 74 (45%) had sold their pigs, and 6 (4%) faced financial constraints that hindered their ability to sustain pig production. The post-intervention survey identified and selected only 317 pigs that met the criteria for sampling.

### 3.2. Baseline KAP and PCC Seroprevalence

The baseline survey showed that 615 (88%) of the 692 farmers interviewed kept between one and ten pigs, and 496 (72%) had heard of PCC. Comparable knowledge, attitudes, and behaviors about PCC were present in both the intervention and control groups from the outset. Only 116 respondents (17%) had good methods for PCC prevention and control, while 502 respondents (72%) had a favorable attitude towards control of PCC. Forty-two per cent (292) of the respondents had little knowledge about PCC.

In addition, the baseline survey showed that the village-level antigen positivity for PCC varied between the two study districts. In Songwe District, the antigen positivity ranged from 5% to 33.3%, while in Kongwa District, it ranged from 0% to 26%. The overall average antigen positivity across the study villages was 9.3%, with an interquartile range (IQR) of 4.6%–14.1%.

**Figure 2:**
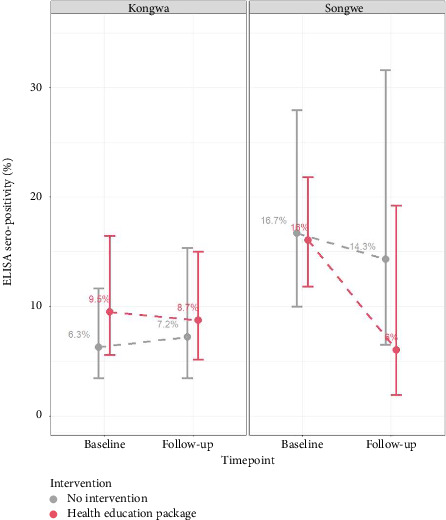
Ag-ELISA positivity between intervention and control villages during baseline and follow-up study in Kongwa and Songwe districts. Note that 1 year after health education, PCC seropositivity reduced by 10% in the intervention villages in Songwe district, while PCC seropositivity reduced by 0.8% in the intervention villages in Kongwa district.

The PCC seropositivity was relatively evenly distributed between the intervention and control villages in both Kongwa and Songwe districts (Figure 2). During the baseline survey, the PCC prevalence in intervention and control villages in Songwe District was 16% (95% CI: 11.8%–21.8%) and 16.7% (95% CI: 10.0%–27.9%), respectively. In Kongwa District, the PCC prevalence in intervention and control villages was 9.5% (95% CI: 5.5%–16.4%) and 6.3% (95% CI: 3.4%–11.6%), respectively.

### 3.3. The Effect of Community-Based HE Intervention on the Enhancement of KAP

The overall change in KAP levels between the intervention and control villages from baseline to follow-up has been assessed. The study found a significant change in KAP levels from baseline to follow-up in both control and intervention groups in Kongwa and Songwe Districts (Table 1). The intervention group showed a greater change in knowledge level compared to the control group, with a significant decrease in low knowledge level (*p* < 0.001) and a significant increase in high knowledge level (*p* < 0.001), Table 1. The intervention villages showed a significant increase in knowledge of transmission during the follow-up period, whereby 78% of pig farmers recognized that pigs acquire *T. solium* cysticercosis through ingesting human feces with *T*. *solium* eggs (*p* < 0.001). The intervention villages also demonstrated a significant improvement in knowledge about preventive measures such as stopping open-field defecation (*p*=0.011) and proper use of latrines (*p*=0.037) (Table 2). The identification of measly pork was correctly described by 35.5% and 41.2% of untrained and trained farmers, respectively, at baseline. The study found significant improvement in knowledge for farmers in both the trained (63.9%) and untrained groups (45.5%) at follow-up (Table 2).

The attitudes in the intervention villages improved significantly, with 88% agreeing that pigs under a free range system are at high risk of acquiring *T. solium* cysticercosis (*p* < 0.001). The intervention villages exhibited a stronger agreement that pork should be inspected by veterinary officials (*p*=0.023) and showed a higher level of willingness to condemn the infected pigs (*p* < 0.001) (Table 2). The intervention villages showed a significant increase in the ability to identify measly pork (*p* < 0.001) and consultation with veterinary officials (*p*=0.002).

### 3.4. Community-Based HE Intervention Effect on Improving Pig Management and Hygiene

Both intervention and control villages demonstrated a significant improvement in the overall latrines and pig pens' qualities with a higher percentage of good-quality pens and latrines in the follow-up period compared to the baseline (*p* < 0.001). In addition, both intervention and control villages showed a significant reduction in open defecation during the follow-up period (*p* < 0.001). However, the intervention villages had slightly higher percentages of no open defecation compared to the control villages. The control villages showed a significant increase in indoor pig management (*p*=0.004) with higher percentages of farmers keeping pigs indoors during the follow-up period. The distribution of pigs in semi-intensive and free-range management didn't show a significant change between the intervention and control villages (Table 3).

Table 4 presents the results of a DID analysis assessing the impact of a community-based HE intervention on participants' KAP related to PCC. The intervention demonstrated a statistically significant improvement in both knowledge (*β* = 1.779, *p*=0.0037) and attitudes over time (*β* = 1.024, *p*=0.038).

Further analysis revealed that sex and district were significant predictors of the outcomes. Male participants exhibited higher knowledge scores (*β* = 1.958, *p* < 0.0001) and more favorable attitudes (*β* = 0.643, *p*=0.009) compared to their female counterparts. Additionally, residing in Kongwa District was strongly associated with elevated knowledge (*β* = 1.947, *p* < 0.0001) and attitude scores (*β* = 3.183, *p* < 0.0001), suggesting a contextual influence on the effectiveness of the intervention.

### 3.5. Seroprevalence of PCC Following Community HE Trial

The seroprevalence of PCC decreased from 11.7% to 7.8% (a reduction of 3.9% points) in villages that received the HE intervention, compared to a marginal decline from 9.7% to 9.4% (0.3% points) in control villages. Disaggregated by district, in Kongwa, intervention villages showed a decrease from 9.5% to 8.7% (−1.2% points), while control villages experienced an increase from 6.3% to 7.2% (+0.9% points). In Songwe, intervention villages showed a substantial drop from 16.0% to 6.0% (−10% points), whereas control villages declined slightly from 16.7% to 15.2% (−1.5% points).

As shown in Table 5 and Figure 2, after adjusting for district-level stratification and village-level clustering, the odds ratio for the overall intervention effect on PCC prevalence was 0.70 (95% CI: 0.27–1.83; *p*=0.47).

## 4. Discussion

This study assessed the effect of a community-based HE intervention using a HEP on KAPs among smallholder pig farmers and the seroprevalence of PCC in disease-endemic settings in Tanzania. The community-based HE intervention was effective in enhancing knowledge and attitude regarding the transmission and prevention of PCC. The findings also suggest that the intervention was more effective over time, which could be due to increasing exposures to HE messages through the given leaflets or a greater understanding of the concepts being taught. This suggests that after the intervention, the community could take the right options on PCC prevention measures, leading to the prevention/control of the *T. solium* parasite. However, the change in practices was not significant and probably required a longer observation period. Research by Kajuna et al. [27] reported a noticeable improvement in the condition of the pig pens and houses about 25 months after the digital HE intervention. This study's findings are consistent with previous studies, which established that HE improves the knowledge of the community on TSTC smallholder pig farmers in various pig diseases [37, 38].

The study found that HE improved farmers' desire to condemn cysticerci-infected pork and to buy/sell pigs or pork that has undergone veterinary inspection. The results of this study align with those reported by Mwidunda et al. [26], which demonstrated that educational interventions positively influenced school children's attitudes toward the condemnation of pigs infected with cysticerci. In Tanzania, the lack of effective treatment options for cysticerci-infected pigs leaves confiscation of contaminated pork as the primary measure to protect public health.

According to the Animal Diseases Act of 2003 and its accompanying Regulations of 2007, pork infected with cysticerci must be fully condemned, and affected pig carcasses disposed of—consistent with the guidelines outlined in the World Organization for Animal Health's Terrestrial Animal Health Code. Although public attitudes toward purchasing or selling pigs and pork subjected to veterinary inspection have become more favorable, practical implementation remains challenging in many rural areas of low- and middle-income countries, including Tanzania. This is largely due to the widespread practice of home-based pig slaughter, where formal meat inspection is typically absent. To address this gap, it is recommended that the Government invest in constructing slaughter facilities at the village level and deploy trained meat inspectors within communities to ensure proper inspection and compliance.

This study found that HE intervention did not affect the disease dynamics in terms of sero-positivity during the observation period. Poor latrine quality, the continued management of semiintensive and free-ranging pigs, and poor pig pens are risk factors that contribute to the transmission of PCC [28, 39], which could partially account for the lack of positive effects of HE intervention. Despite the observed increase in pig confinement during follow-up, some pigs could still access human feces in the contaminated environment due to partial pig confinement and open-field defecation behavior. In both the intervention and the control villages, the study observed a general reduction in the prevalence of PCC after the intervention, indicating that the reduction cannot be due to the intervention alone but rather may be the consequence of other reasons that took place in the study areas such as sanitation initiatives (the house is toilets) that were launched since 2018 and focused on encouraging households to build and utilize latrines hygienically. The campaign might be the reason for PCC reduction due to a decrease in reported open-field defecation behavior as resulted in a decrease in the environmental contamination with human feces, hence reducing the environmental contamination with the parasite eggs. Another reason could be the COVID-19 pandemic, which was first reported in Tanzania in February 2020. As a means of protection from the pandemic, frequent hand washing was emphasized. Hand washing is one of the ways to prevent/control *T. solium* infections [40]. The results of this study are comparable to those of a study by Kajuna et al. [27], in which a digital health literacy intervention had no appreciable effect on PCC prevalence.

This study found that there was a significant improvement in the “observed” practices, such as indoor pig management, good pig pens, and quality latrines in both intervention and control villages. The changes observed in both the control and intervention villages suggest that the improvement was not due to the HE alone, but rather to other factors such as information contamination from the intervention villages or other sources or like the COVID-19 pandemic, enforcement of bylaws governing indoor pig rearing, sanitation initiatives, or the Hawthorne effect. The Hawthorne effect is a type of human behavior reactivity that occurs when people alter one aspect of their behavior when they become aware that they are being observed [16]. Change in practices may be influenced by poverty and by cultural norms, and therefore, difficult to change in a short time. So, to observe positive behavioral changes, more time is needed. This study's findings are comparable to those of earlier ones that were carried out in Tanzania [26] and Mozambique [38].

### 4.1. The Study's Benefits and Shortcomings

The study's strength is in the cluster design, which, takes into account, is important for assessing the intervention's effect on the follow-up response based on the baseline response. Also, the study used the developed HEP from community participation. The mode of delivery of HE was designed to ensure the sustainability of HE in the study area beyond the study period. The current research has some limitations. HE has some drawbacks as a stand-alone strategy, primarily because learning does not always result in behavioral and practical changes needed to reduce disease incidence. As a result, cross-disciplinary approaches are anticipated in the mediation. In addition, unlike other intervention tools, we have not included a practical section on pig feeding and housing structure. In addition, this study did not include an assessment and treatment of human tapeworm carriers, which would be an important step to immediately interrupt the life cycle of *T. solium,* which may have helped see larger short-term effects of our HE intervention. Furthermore, another potential limitation of this study is that estimated seroprevalence of PCC relies solely on Ag-ELISA, which may either underestimate or overestimate the true prevalence due to the test's diagnostic inaccuracies. Although the assay is highly sensitive, it cannot differentiate between species such as *T. solium* and *T. hydatigena*. Recently, the sensitivity and specificity of the test were reported to be 82.7% and 86.3%, respectively [33]. The study may have estimated the prevalence of *T. hydatigena*, which might have contributed to cross-reactions and inflated seroprevalence estimates. This emphasizes the need for future studies to use accurate diagnostic tools that can differentiate between *Taenia* species in pigs. Hence, we provide more precise seroprevalence estimates and strengthen the foundation for designing effective intervention strategies.

## 5. Conclusions

This study revealed that community-based HE intervention using the HEP has significantly improved the knowledge and attitudes of smallholder pig farmers toward PCC transmission and control. Although a HEP improved people's KAP and contributed to reducing PCC prevalence, it is insufficient as a standalone intervention to achieve sustainable control of *Taenia solium*. An integrated approach through a One Health framework is essential, incorporating interventions that address both the intermediate and final hosts, alongside environmental and behavioral factors, for effective management of *Taenia solium* in endemic regions.

## Figures and Tables

**Table 1:** Change in knowledge, attitudes, and practice levels from baseline to follow-up in Kongwa and Songwe districts 2019–2022 (*n* = 486).

	**Control group**				**Intervention**			
	**Baseline**	**Follow-up**	**Changes in control group (%)**	**p** **-value**	**Baseline**	**Follow-up**	**Change in intervention group (%)**	**p** **-value**
	***N* = 231**	***N* = 255**			***N* = 231**	***N* = 255**		
	***n*, %**	***n*, %**			***n*, %**	***n*, %**		

*Change in knowledge level from baseline to follow-up*								
Low	102 (44.2)	101 (43.7)	−0.5	0.930	94 (36.9)	50 (19.6)	−17.3	< 0.001^∗∗^
Moderate	105 (45.5)	115 (49.8)	4.3	0.350	128 (50.2)	173 (67.8)	17.6	< 0.001^∗∗^
High	24 (10.4)	15 (6.5)	−3.9	0.130	33 (12.9)	32 (12.5)	−0.4	0.89

*Change in attitude level from baseline to follow-up*								
Negative	45 (19.5)	32 (13.9)	−5.6	0.100	24 (9.4)	19 (7.5)	−1.9	0.430
Neutral	20 (8.7)	30 (13.0)	4.3	0.130	32 (12.5)	8 (3.1)	−9.4	< 0.001^∗∗^
Positive	166 (71.9)	169 (73.2)	1.3	0.750	199 (78.0)	228 (89.4)	11.4	< 0.001^∗∗^

*Change in practice level from baseline to follow-up*								
Control	*N* = 231	*N* = 231						
Poor	90 (39.0)	128 (55.4)	16	< 0.001^∗∗^	98 (38.4)	108 (42.4)	4	0.370
Moderate	88 (38.1)	84 (36.4)	−1.7	0.70	92 (36.1)	106 (41.6)	5.5	0.200
Good	53 (22.9)	19 (8.2)	−14.7	< 0.001^∗∗^	65 (25.5)	41 (16.1)	−9.4	< 0.001^∗∗^

*Note: N*, number of pig farmers.

^∗^Significant (*p* < 0.05).

^∗∗^Significant (*p* < 0.001).

**Table 2:** Change in pig farmer's knowledge attitudes and reported practices between intervention and control villages from baseline to follow-up post–health education in Kongwa and Songwe districts, Tanzania 2019–2022 (*n* = 486).

Variables	Control villages				Intervention villages			
Questions related to knowledge	Baseline *N* = 231	Follow-up *N* = 231	Change in control group	*p*-value	Baseline *N* = 231	Follow-up *N* = 231	Change in intervention group	*p* value
	*n*, %	*n*, %	(%)		*n*, %	*n*, %	(%)	
*How does T. solium cysticercosis spread among pigs??*								
Consuming *T. solium* eggs through human feces	67 (29.0)	93 (40.3)	11.3	0.011^∗^	93 (36.5)	199 (78.0)	41.5	< 0.001^∗∗^
Is it possible to stop pigs from developing T. *solium cysticercosis*? (Yes)	98 (42.4)	104 (45.0)	2.6	0.570	116 (45.5)	202 (79.2)	34.0	< 0.001^∗∗^

*If so, describe the precautions*								
Pigs being housed indoors all the time	92 (93.9)	104 (100.0)	6.1	0.010^∗^	114 (98.3)	202 (100.0)	1.7	0.061
Proper use of toilets	2 (2.0)	7 (6.7)	4.7	0.790	3 (2.6)	35 (17.3)	14.7	0.011^∗^
People should quit defecating in open spaces	11 (11.2)	3 (2.9)	−8.3	0.011^∗^	3 (2.6)	19 (9.4)	6.8	0.037^∗^

*Questions related to attitudes*								
Free-range pigs are at high risk of acquiring *T. solium* cysticercosis (Agree)	133 (57.8)	160 (69.3)	11.5	0.400	157 (61.6)	225 (88.2)	26.6	< 0.001^∗∗^
I must only purchase or sell pork that has undergone inspection by veterinary officials. (Agree)	175 (75.8)	204 (88.3)	12.5	0.220	205 (80.4)	240 (94.1)	13.7	0.023^∗^
I would abhor pork infected with *T. solium* cysts.	143 (61.9)	151 (65.4)	3.5	0.034^∗^	181 (71.0)	218 (85.5)	14.5	< 0.001^∗∗^

*Reported practices*								
Can you identify measly pork? (Yes)	82 (35.5)	105 (45.5)	10	0.029^∗^	105 (41.2)	163 (63.9)	22.7	< 0.001^∗∗^

*Which actions would you take if you found your pigs are infected with T. solium cysts?*								
Consult veterinary doctor	128 (55.4)	139 (60.2)	4.8	0.300	163 (63.9)	195 (76.5)	12.6	0.002^∗^
Wash your hands before preparing pig feed (Yes)	58 (25.1)	36 (24.3)	−0.8	0.480	57 (22.4)	55 (32.4)	10	0.110

*Treatment of water before drinking*								
Boil	4 (1.7)	30 (13.0)	11.3	0.140	1 (0.4)	40 (15.7)	15.3	0.400

*Note: N*, number of pig farmers.

^∗^Significant (*p* < 0.05).

^∗∗^Significant (*p* < 0.001).

**Table 3:** Change in pig farmer's pig management and the hygienic situation between intervention and control villages from baseline to follow-up post–health education in Kongwa and Songwe districts, Tanzania 2019–2022.

Variables	Control villages			Intervention villages		
	Baseline *N*, %	Follow-up *N*, %	*p* value	Baseline *N*, %	Follow-up *N*, %	*p* value
*Pig management*						
Indoor	171 (76.7)	136 (85.5)	0.041^∗^	197 (81.4)	135 (83.3)	0.650
Semiintensive	34 (15.2)	11 (6.9)		26 (10.7)	13 (8.0)	
Free-range	18 (8.1)	12 (7.5)		19 (7.8)	14 (8.6)	

*Overall latrine impression*						
Good	16 (7.5)	51 (22.7)	< 0.001^∗∗^	12 (5.3)	55 (24.2)	< 0.001^∗∗^
Moderate	162 (76.1)	125 (55.6.)		189 (83.3)	126 (55.5)	
Poor	35 (16.4)	49 (21.8)		26 (11.5)	46 (20.3)	

*Overall pig pen impression*						
Good	17 (8.9)	24 (17.0)	0.001^∗∗^	12 (5.8)	37 (25.9)	< 0.001^∗∗^
Moderate	145 (75.5)	78 (55.3)		173 (83.2)	85 (59.4)	
Poor	30 (15.6)	39 (27.7)		23 (11.1)	21 (14.7)	

*Presence of drinkers in the pig pen?*						
No	67 (30.3)	53 (37.6)	0.152	114 (47.3)	68 (47.6)	0.962
Yes	154 (69.7)	88 (62.4)		127 (52.7)	75 (52.4)	

*Presence of feeders in the pig pen?*						
No	30 (15.5)	17 (12.1)	< 0.001^∗∗^	43 (20.7)	4 (2.8)	< 0.001^∗∗^
Yes	163 (84.5)	124 (87.9)		165 (79.3)	139 (97.2)	

*Open defecation by children?*						
No	158 (72.1)	236 (99.2%)	< 0.001^∗∗^	162 (69.5)	243 (100.0)	< 0.001^∗∗^
Yes	61 (27.9)	2 (0.8)		71 (30.5)	0 (0.0)	

*Note: N*, number of pig farmers.

^∗^Significant (*p* < 0.05).

^∗∗^Significant (*p* < 0.01).

**Table 4:** Community health education effect on knowledge, attitudes, and practices towards porcine cysticercosis in Kongwa and Songwe districts, Tanzania 2019–2022 (*n* = 486): difference-in-difference (DID) estimation.

Effect	Knowledge			Attitude			Practices		
	Estimate (*β*)	Standard error	*p* value	Estimate (*β*)	Standard error	*p*-value	Estimate (*β*)	Standard error	*p* value
Intervention	1.508	0.451	< .001^∗∗^	1.229	0.283	< 0.001^∗∗^	0.457	0.224	0.042^∗^
Control	Reference			Reference					
Time ∗ Treatment	1.779	0.609	0.004^∗∗^	1.0244	0.492	0.038	0.719	0.317	0.023^∗^
Sex									
Male	1.958	0.344	< 0.001^∗∗^	0.643	0.247	0.001^∗∗^	−0.067	0.179	0.709
Female	Reference			Reference					
District									
Kongwa	1.947	0.366	< 0.001^∗∗^	3.183	0.253	< 0.001^∗∗^	2.501	0.193	< 0.001^∗∗^
Songwe	Reference			Reference					
Age									
15–25	Reference			Reference					
26–35	1.245	0.624	0.047^∗^	0.922	0.439	0.036	0.798	0.323	0.014^∗^
36–45	1.787	0.622	0.004^∗∗^	1.477	0.441	< 0.001^∗∗^	1.442	0.323	< 0.001^∗∗^
46–55	2.066	0.656	0.002^∗∗^	1.758	0.465	< 0.001^∗∗^	1.242	0.341	< 0.001^∗∗^
56+	2.245	0.713	0.002^∗∗^	1.896	0.508	< 0.001^∗∗^	1.071	0.371	0.004^∗∗^
Educational attainment									
Without a formal education	Reference			Reference					
Primary Education	2.333	0.407	< 0.001^∗∗^	1.736	0.288	< 0.001^∗∗^	1.267	0.211	< 0.001^∗∗^
Secondary and above	4.428	0.646	< 0.001^∗∗^	2.989	0.458	< 0.001^∗∗^	3.113	0.337	< 0.001^∗∗^

*Note: N*, number of pig farmers.

^∗^Significant (*p* < 0.05).

^∗∗^Significant (*p* < 0.01).

**Table 5:** Prevalence of PCC following TSTC community-based health education trial in Kongwa and Songwe districts in Tanzania.

Variables	Baseline		Follow-up		Change	Odds ratio [95%CI]£
	*n*/*N*	Prevalence (%) [95%CI]∗	*n*/*N*	Prevalence (%) [95%CI]∗	Prevalence (%)	
Health education intervention	37/353	11.7 (8.3–16.5)	12/165	7.8 (4.7–13.1)	−3.9	0.70 [0.27–1.83]
Kongwa	20/250	9.5 (5.5–16.4)	9/121	8.7 (5.1–14.9)	−0.8	
Songwe	17/136	16.0 (11.8–21.8)	6/54	6.0 (1.9–19.2)	−10.0	
No intervention	30/339	9.7 (6.4–14.8)	13/152	9.4 (5.3–16.5)	−0.3	
Kongwa	13/233	6.3 (3.4–11.6)	7/111	7.2 (3.4–15.3)	+0.9	
Songwe	17/140	16.7 (10–27.9)	3/56	15.2 (6.5–31.6)	−1.5%	

*Note:* £ Odds ratio for the intervention effect; 95%CI: 95% confidence interval; *n* = PCC seropositive pig, *N* = number of pigs examined.

^∗^Prevalence taking into account the study design (clustering on village level and strata by district).

## Data Availability

The data supporting the findings of this study are available from the corresponding author upon reasonable-request.
